# Regulatory Effects of Tannin Supplementation on Microbial Succession and Flavor Formation During Xiaoqu Light-Flavor Baijiu Fermentation

**DOI:** 10.3390/foods15162833

**Published:** 2026-08-14

**Authors:** Siyu Li, Xiao Yu, Huiling Huang, Chenyang Wang, Chun Yi, Xinying Zhang, Yong Wen, Bing Xiong, Qingshan Jiang, Kangjie Yu, Yi Ma

**Affiliations:** 1 Sichuan Province Engineering Technology Research Center of Liquor-Making Grains, School of Food and Liquor Engineering, Sichuan University of Science and Engineering, Yibin 644000, China; 324095102420@stu.suse.edu.cn (S.L.); 322095102432@stu.suse.edu.cn (X.Y.); 322086002325@stu.suse.edu.cn (H.H.); 324095102429@stu.suse.edu.cn (C.W.); 324086002325@stu.suse.edu.cn (C.Y.); 324086002333@stu.suse.edu.cn (X.Z.); 2 Sichuan Provincial Academy of Natural Resource Sciences, Chengdu 610015, China; 3 Yibin Grain and Oil Industry Development Center, Yibin 644000, China; shenmu1@foxmail.com; 4 Sichuan Provincial Alcoholic Beverage Research and Development Center, Chengdu 610095, China; 18382547494@163.com; 5 Yibin Agricultural Science and Technology Research Institute, Yibin 644000, China; 15881382610@163.com; 6 College of Agronomy, Northwest A&F University, Yangling 712100, China

**Keywords:** tannins, Xiaoqu light-flavor Baijiu, microbial succession, volatile flavor compounds, co-occurrence network

## Abstract

Sorghum tannins have been suggested to influence microbial ecology and flavor formation in Xiaoqu light-flavor Baijiu (XLB), but their specific contribution is difficult to distinguish from confounding, cultivar-dependent variations in macromolecular components. To address this limitation, a controlled fermentation system was established using a uniform, low-tannin substrate. Based on preliminary gradient trials, a 1.0% tannin supplementation level was selected, and high-throughput sequencing combined with HS-SPME-GC-MS was employed to investigate tannin-related microbial and volatile changes. Compared with the group without tannin supplementation, 1.0% tannin supplementation altered bacterial and fungal community succession during the fermentation, reducing the relative abundances of *Saccharomyces* and *Weissella*, and enriching taxa including *Cyberlindnera* and *Pantoea*. The tannin-supplemented group exhibited a more complex and stable microbial co-occurrence network. Furthermore, PICRUSt2 predictions suggested enhanced metabolic potentials primarily related to carbohydrate and amino acid pathways. FUNGuild analysis further suggested that tannin supplementation shifted fungal trophic-mode composition, particularly saprotrophic and saprotroph-containing groups. At the end of fermentation, total volatile compounds increased from 4.208 μg/g to 4.983 μg/g, total esters and acids increased by 27.0% and 112.3%, respectively, whereas total alcohols decreased by 13.3%. Spearman correlation analysis revealed that the enriched non-*Saccharomyces* fungi and acid-producing bacteria in the tannin-supplemented group were positively associated with the accumulation of acids and esters, whereas the control microbiota was mainly linked to an alcohol-oriented profile. These findings may provide process-level evidence for tannin-related microbial and volatile changes during XLB fermentation.

## 1. Introduction

Xiaoqu light-flavor Baijiu (XLB), primarily produced from sorghum and characterized by its clean flavor and short fermentation cycle, occupies a pivotal position in the Chinese distilled spirits market [1,2,3]. As the fundamental substrate in jiupei (fermented grains) fermentation, sorghum provides essential carbon and nitrogen while also constituting a complex matrix that shapes both microbial community assembly and flavor compound formation [4,5,6]. Consequently, deciphering how specific raw material components modulate this micro-ecological and flavor system is critical for precision quality control.

In this regard, considerable attention has been given to exploring the roles of sorghum’s macromolecular constituents, such as starch, protein, and lipids, which have been widely acknowledged as essential drivers of the fermentation process [7]. These investigations have elucidated that starch properties govern glucose availability and fermentation efficiency, whereas protein and lipid contents act as the primary modulators of flavor precursors, directly influencing the typicity of Baijiu [8,9,10]. Building on these insights into macronutrients, recent research has pivoted to the profound influence of micro-components, including organic acids, metal ions, and phenolics, revealing their effects on both fermentation dynamics and the flavor profile of Baijiu [11,12,13]. Similar phenomena in other fermented beverages (e.g., wine and beer) indicate that tannins broadly influence microbial selection, enzyme activity, and flavor expression, underscoring their role as a general ecological and sensory factor in fermentation [14,15,16]. As a predominant class of phenolic compounds, tannins are critical modulators of microbial physiological states and flavor nuances in Baijiu, despite their relatively low concentration in sorghum [17,18]. Tannins are broadly categorized into two main classes—condensed and hydrolyzable—with sorghum tannins being predominantly of the condensed type [19]. During fermentation and distillation, tannins undergo partial degradation into phenolic and aromatic substances that directly contribute a desirable bitterness and complexity to Baijiu’s flavor [7]. Simultaneously, they can bind proteins and enzymes through hydrogen bonding and hydrophobic interactions to modulate saccharification and selectively inhibit microbial growth, thereby indirectly shaping the spectrum of volatile flavor compounds produced [20,21,22]. Lu et al. [23] revealed that specific tannin content contributes to the aromatic complexity of distilled spirits, while Zhu et al. [24] demonstrated that sorghum cultivars with varying tannin profiles significantly drive microbial community succession and flavor metabolism in Light-flavor and Strong-flavor Baijiu, respectively.

However, a critical methodological limitation persists in these investigations. Most studies rely on comparative research between different sorghum varieties, which inevitably introduces confounding variables, such as divergent genetic backgrounds, distinct starch structures, and varying protein contents. As a result, the observed differences in microbial succession or flavor metabolism cannot be unequivocally attributed to tannin alone. For example, studies based on glutinous and non-glutinous sorghum varieties have suggested that high tannin content inhibits *Saccharomyces cerevisiae*, especially strains pivotal for light-flavor Baijiu [25]; yet, such comparisons fail to isolate the specific effect of tannins from other compositional differences. Moreover, in studies such as the investigation of waxy characteristics by Ren et al. [26] and the comparative sensomics analysis by Ma et al. [5], the potential regulatory effects of tannins are inevitably overshadowed by or confounded with variations in macronutrient profiles. This methodological constraint makes it challenging to decouple and better evaluate the contribution of tannins from the overall genetic background of the sorghum grain. This limitation weakens causal interpretation and restricts the ability to clarify whether tannins independently reshape microbial assembly, metabolic function, and flavor generation during fermentation.

To address this limitation, tannin supplementation was used as a controlled strategy to evaluate the specific contribution of tannins under a uniform sorghum substrate background, minimizing confounding effects caused by cultivar-dependent variations in macromolecular components. Tannins were extracted and purified, and a representative supplementation level was selected through preliminary gradient experiments. In these trials, tannin supplementations of 0, 0.5, 1.0, 1.5, and 2.0% were evaluated, revealing that a 1.0% tannin supplementation significantly mitigated harsh-tasting compounds and elevated the concentrations of esters linked to floral and fruity flavor in the base liquor, thereby shifting the sensory profile toward enhanced floral and sweet notes [27]. Based on this result, 1.0% tannin was introduced into a controlled fermentation system in the present study. We hypothesized that tannin supplementation at this level would influence microbial succession and interaction patterns, modify predicted metabolic functions, and thereby alter volatile flavor formation during fermentation. Accordingly, this study investigated the effects of 1.0% tannin on bacterial and fungal communities, key differential taxa, volatile flavor compounds, and functional potential, to clarify the specific role of tannins in influencing microbial succession and flavor formation during XLB fermentation and to support the rational utilization of tannin in XLB production.

## 2. Materials and Methods

### 2.1. Materials

Australian red sorghum (Xike Agriculture Group, Mianyang, China) contained 0.2% tannin (GB/T 15686-2008 [28]), 70.3% starch (GB 5009.9-2023 [29]), 10.5% protein, 3.0% lipid, and 14.1% moisture (GB 5009.3-2016 [30]). Xiaoqu (Huaxi Jiuqu Factory, Chengdu, China) was used. Using Yibin glutinous red sorghum as the raw material, the extraction and purification of sorghum tannin were carried out according to the method of Stalikas [31]. The purity of sorghum tannin was determined and calculated using the vanillin-HCl method, and the purity of sorghum tannin was greater than 95%. This method estimates total condensed tannins; thus, reported purity is a relative value, not an absolute compositional measure. Experimental setups involved supplementing the sorghum with tannin at 0% (TN0_0) and 1.0% (TN1_0) (*w*/*w*) before the steaming process, followed by homogenization.

After manual cleaning to remove stones and weeds, 1 kg of sorghum was placed in a stainless-steel container. Boiling water was added with continuous stirring until the temperature dropped to 45 °C, then the water was discarded. The sorghum was transferred into a cloth bag and immersed in hot water at 50 °C for 12 h. After draining, the grains were evenly spread on a gauze-lined steaming basket and steamed without the lid for 10 min, followed by 20 min with the lid on. The grains were immediately transferred into 45 °C water for 30 min to allow full expansion, then returned to the steamer for a final steaming with the lid closed. Meanwhile, 50 g of rice husks were washed and steamed separately for 30 min. After final steaming, the sorghum was cooled to 30 °C, inoculated with Xiaoqu starter, and mixed. The steamed rice husks were spread over the sorghum surface, covered with eight layers of gauze, and incubated at 26 °C (0–24 h), 30 °C (24–36 h), and 33 °C (36–48 h). After incubation, the sorghum and rice husks were thoroughly mixed and transferred to pottery jars for fermentation at 30 °C. Jiupei samples (50 g) were aseptically collected on days 0, 3, 6, and 10 and stored at –80 °C. Fermentations were carried out in triplicate as independent replicates for each treatment. Samples collected from each batch at each time point were analyzed separately.

### 2.2. DNA Extraction

Total genomic DNA was extracted from jiupei samples using the DNeasy PowerSoil Kit (QIAGEN, Hilden, Germany) according to the manufacturer’s protocols. The quality and integrity of the extracted DNA were verified by 1% agarose gel electrophoresis. DNA quality was initially assessed by 1% agarose gel electrophoresis, and DNA concentration was determined using NanoDrop2000. The DNA concentrations of all samples ranged from 2.80 to 7.20 ng/μL, with most samples between 4.0 and 5.0 ng/μL. The OD260/280 values were generally >1.8, indicating acceptable DNA purity for downstream PCR amplification. The bacterial 16S rRNA V3-V4 hypervariable region, which provides broad bacterial coverage and taxonomic resolution commonly used in fermentation microbiome, was amplified from the metagenomic DNA using the universal primer pair 515F (5′-GTGCCAGCMGCCGCGG-3′) and 806R (5′-GGACTACHVGGGTWTCTAAT-3′). Similarly, the fungal ITS1 region was amplified with the primers ITS1F (5′-CTTGGTCATTTAGAGGAAGTAA-3′) and ITS2R (5′-GCTGCGTTCTTCATCGATGC-3′).

### 2.3. High-Throughput Sequencing Data Analysis

The paired-end raw reads were quality-controlled using fastp (version 0.19.6) and assembled with FLASH (version 1.2.11). Specifically, reads were trimmed using a quality threshold of 20 within a 50 bp sliding window. Reads shorter than 50 bp after trimming or containing ambiguous nucleotides were discarded. Paired reads were merged with a minimum overlap of 10 bp and a maximum mismatch ratio of 0.2 in the overlap region. Demultiplexing was performed based on exact matching to barcodes and allowing up to 2 mismatches in primer sequences.

Amplicon sequence variants (ASVs) were inferred from the quality-filtered sequences using the DADA2 plugin [32] within the QIIME2 pipeline [33], following default parameters. All sequences annotated as chloroplasts or mitochondria were removed from the dataset. To minimize the influence of sequencing depth on downstream alpha- and beta-diversity analyses, all samples were rarefied to 20,000 sequences per sample. Following rarefaction, the average sequence coverage across samples, as measured by Good’s coverage, remained high at 99.09%. Taxonomic assignment of ASVs was performed using the Naive Bayes classifier in QIIME2 against the SILVA 16S rRNA gene database (release 138). Functional potential of the bacterial communities was predicted only using PICRUSt2 (version 2.2.0) [34]. All high-throughput sequencing experiments were conducted by Shanghai Majorbio Bio-pharm Technology Co., Ltd. (Shanghai, China).

### 2.4. Determination of Volatile Flavor Compounds

Volatile flavor compounds in the jiupei samples were analyzed by headspace solid-phase microextraction coupled with gas chromatography-mass spectrometry (HS-SPME-GC-MS) using an Agilent 7890A gas chromatograph (Agilent, Santa Clara, CA, USA). Sample preparation was performed according to a previously described method [35] with minor modifications. Briefly, 4 g of jiupei was homogenized with 10 mL of physiological saline and soaked overnight at 4 °C. After ultrasonic extraction for 30 min, the mixture was centrifuged at 10,000 rpm for 25 min. Then, 5 mL of the supernatant was transferred to a 15 mL headspace vial, mixed with 1.5 g of NaCl and 20 μL of 2-octanol (0.2 g/L) as an internal standard. The vial was incubated in a preheated SPME system at 50 °C for 5 min for equilibrium, followed by extraction with an SPME fiber for 45 min.

The analysis was performed on a GC system equipped with a DB-WAX capillary column (60.0 m × 0.25 mm × 0.25 µm, Agilent) and a mass spectrometric detector. The injector temperature was set at 250 °C in splitless mode. The oven temperature program was as follows: held at 50 °C for 2 min, increased to 230 °C at a rate of 4 °C/min, and finally held for 5 min. High-purity helium (99.999%) was used as the carrier gas at a constant flow rate of 1.0 mL/min. The mass spectrometer was operated in electron ionization (EI) mode with an ion source temperature of 230 °C and an electron energy of 70 eV. The quadrupole temperature was maintained at 100 °C, and the mass scan range was set from 35 to 400 amu. Volatile compounds were tentatively identified by comparing their mass spectra with those in the NIST11.L database, retaining only matches with a similarity score greater than 80%. Further confirmation was achieved by comparing the calculated retention indices (RIs) of the compounds with the reference RIs available in the NIST Chemistry WebBook (https://webbook.nist.gov/chemistry, accessed on 16 May 2024). Semi-quantification of the volatile compounds was conducted based on the peak area ratio of each compound to the internal standard (2-octanol). This semi-quantitative approach was used to compare relative volatile profiles among samples, making the data suitable mainly for relative comparison between treatments rather than absolute quantification.

### 2.5. Odor Activity Value Calculation

To preliminarily assess the potential odor contribution of individual volatile compounds detected in jiupei, odor activity values (OAVs) were calculated according to the following formula:

(1) $$\mathrm{OAV}=C_{i}/T_{i}$$

where $C_{i}$ represents the semi-quantitative concentration of volatile compound i in the jiupei sample (expressed as µg/g), and $T_{i}$ is its odor threshold in aqueous solution or an ethanol–water matrix as reported in the literature. Compounds with OAV > 1 were considered to potentially contribute directly to the overall aroma of the jiupei at the detected levels. Given that the concentration data were obtained from semi-quantitative HS-SPME-GC–MS analysis and that the referenced thresholds were determined in matrices different from jiupei, the calculated OAVs serve only as a preliminary indication of odor relevance. Odor threshold values were primarily sourced from the compilations by van Gemert [36], Fenaroli’s Handbook of Flavor Ingredients [37] and the related literature [38].

### 2.6. Statistical Data Analysis

All experimental data are presented as the mean ± standard deviation from three independent replicates. For endpoint data collected at the end of fermentation (day 10), comparisons between the two groups were performed using Student’s *t*-test in SPSS software 30.0, with a *p*-value < 0.05 considered statistically significant. Temporal changes in microbial community composition were described using sequencing-derived relative abundance profiles. Because these compositional data are constrained by total community structure, individual taxa were not treated as independent continuous variables for extensive significance testing across fermentation time [39]. Principal coordinate analysis (PCoA) based on Bray–Curtis distance and linear discriminant analysis effect size (LEfSe) for differential species identification were performed and visualized on the Majorbio Cloud Platform (https://cloud.majorbio.com/page/tools.html, accessed on 30 April 2024). Both were conducted using the default workflow of the platform, and taxa with LDA scores > 3.5 were considered differentially abundant. All analyses were conducted using R software (version 4.4.1). Co-occurrence networks were constructed using the igraph package (v1.2.6) in R to determine relationships among microorganisms within the community, and the networks were visualized using Gephi 0.10.1 (Web Atlas, Paris, France). Within-module connectivity (Zi) and among-module connectivity (Pi) were calculated using R to identify key taxa. Correlation heatmaps were generated using the GenesCloud platform (https://www.genescloud.cn/home) and the Wekemo Bioincloud (https://www.bioincloud.tech). The metabolic functional potential of bacterial communities was predicted using PICRUSt2 based on the KEGG database [40]. Fungal trophic modes were annotated in R using FUNGuildR against the FUNGuild database (http://www.stbates.org/guilds/app.php, accessed on 2 Augest 2026).

## 3. Results and Discussion

### 3.1. The Composition and Differences in Microbial Communities

The jiupei in Xiaoqu light-flavor Baijiu production represents a complex ecosystem that sustains a rich microbiota, which is both the main driver of the brewing process and the origin of flavor compounds [41]. Through high-throughput sequencing and high-resolution ASV clustering analysis of the jiupei microbiota, we delineated the composition and diversity of the microbial communities in TN0_0 and TN1_0.

#### 3.1.1. Microbial Diversity

**Figure 1:**
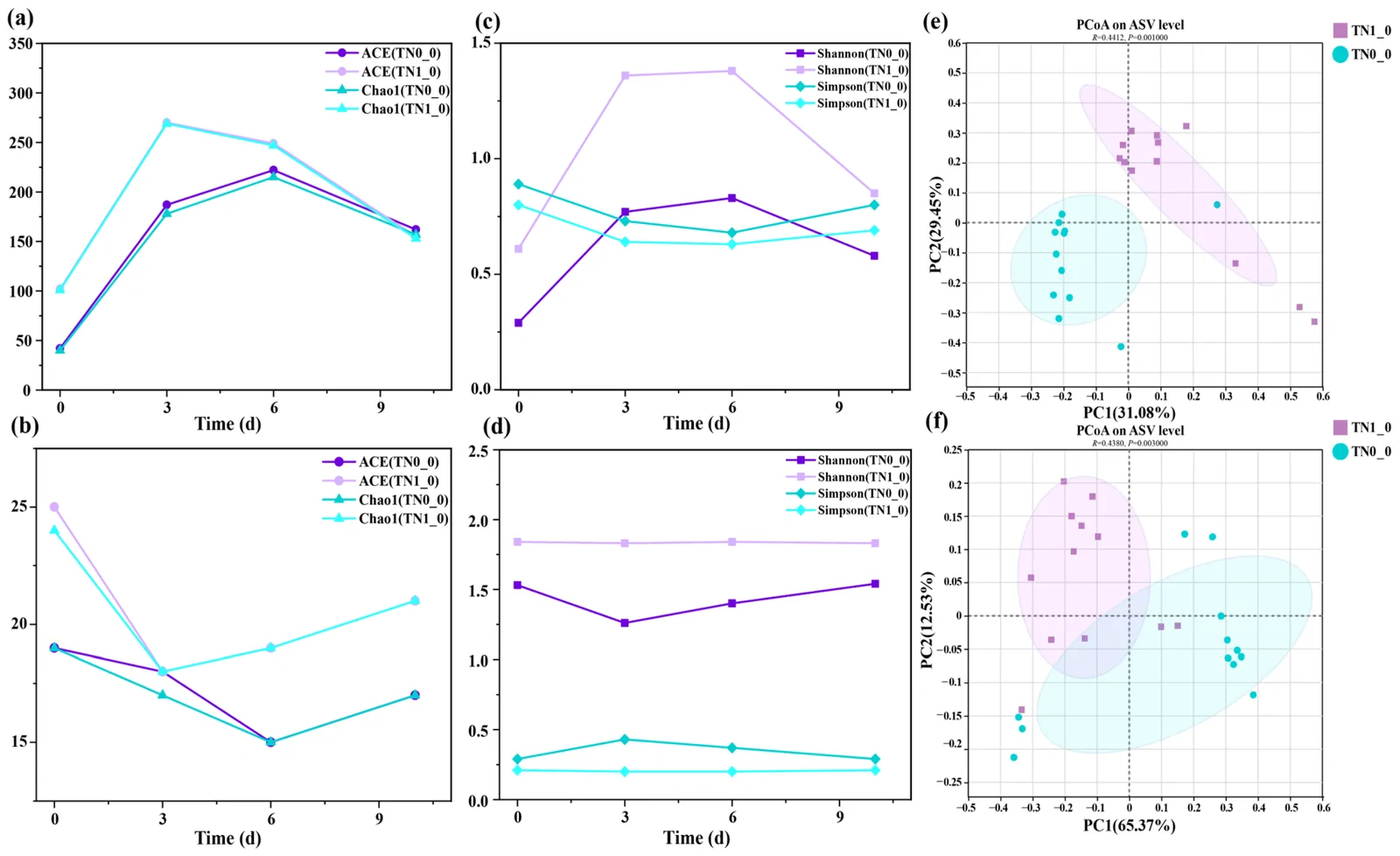
Microbial diversity of bacterial and fungal communities in XLB jiupei from TN0_0 and TN1_0 groups at days 0, 3, 6, and 10. ACE and Chao1 indices for bacterial (**a**) and fungal (**b**) communities. Shannon and Simpson indices for bacterial (**c**) and fungal (**d**) communities. Principal coordinate analysis (PCoA) based on Bray–Curtis distance for bacterial (**e**) and fungal (**f**) communities, with the percentage of variance explained by each principal coordinate indicated on the axes.

Species richness was assessed using the ACE and Chao1 indices, which estimate the total taxonomic richness in a community, with a particular consideration for rare species [42]. The ACE and Chao1 indices of the bacterial community were higher in TN1_0 at most fermentation time points, although a slight reversal was observed on day 10 (Figure 1a). During the fermentation process, the ACE and Chao1 indices of the fungal communities in the two groups of jiupei changed minimally and showed only minor fluctuations (Figure 1b). It can be suggested that the supplementation of 1.0% tannin was generally associated with increased bacterial richness during most fermentation stages, whereas its effect on the fungal community was limited. A similar divergence between bacterial and fungal α-diversity has been reported during light-flavor Baijiu fermentation using different sorghum varieties, in which the sorghum type associated with higher tannin content exhibited higher bacterial α-diversity, whereas fungal diversity followed a different pattern [6]. However, because sorghum varieties also differ in starch structure, protein, lipids, and other components, this similarity should be regarded as supportive rather than tannin-specific evidence. The limited response of the fungal community may be related to the higher tolerance or ecological stability of dominant fungi under the fermentation conditions.

Microbial diversity was evaluated using the Shannon and Simpson indices, which correlate positively and inversely with diversity, respectively [43]. Increased Shannon and decreased Simpson indices in the TN1_0 across all time points indicated a consistently higher α-diversity pattern compared with TN0_0 (Figure 1c,d). Therefore, tannin supplementation appears to positively affect species diversity in jiupei.

Differences in bacterial and fungal community structures between the two groups during fermentation were assessed by Principal Coordinate Analysis (PCoA) based on Bray–Curtis distance (Figure 1e,f). The ANOSIM method was used for the inter-group difference test. The results showed that there were extremely significant differences between the two groups of jiupei at different fermentation times (*p* < 0.01). For bacterial communities, a significant separation was observed between TN1_0 and TN0_0 (*R* = 0.4412, *p* = 0.001). The first principal coordinate (PC1) explained 31.08% of the variance, indicating that tannin supplementation was a major driver of bacterial community variation, which might imply an associated shift in metabolic functional potential. The effect of tannin supplementation on the fungal community was even more pronounced, showing a strong and clustered separation (*R* = 0.4380, *p* = 0.003). Notably, PC1 alone accounted for 65.37% of the total variance, indicating that the main ordination axis captured a large proportion of the variation in fungal community composition.

Taken together, the results indicate that tannin supplementation was associated with shifts in bacterial and fungal community structures during jiupei fermentation.

#### 3.1.2. Microbial Community Composition

While the divergence in alpha and beta diversity indices confirmed the potent role of tannins in altering the community equilibrium, the specific compositional shifts underlying these structural changes remained to be clarified. Thus, we further tracked the temporal dynamics of dominant microbial taxa throughout the fermentation cycle based on relative abundance profiles. The temporal microbial composition results were interpreted mainly on the basis of relative abundance patterns derived from sequencing data. These results were used to describe community succession trends rather than to support extensive significance testing of individual taxa across the fermentation process.

**Figure 2:**
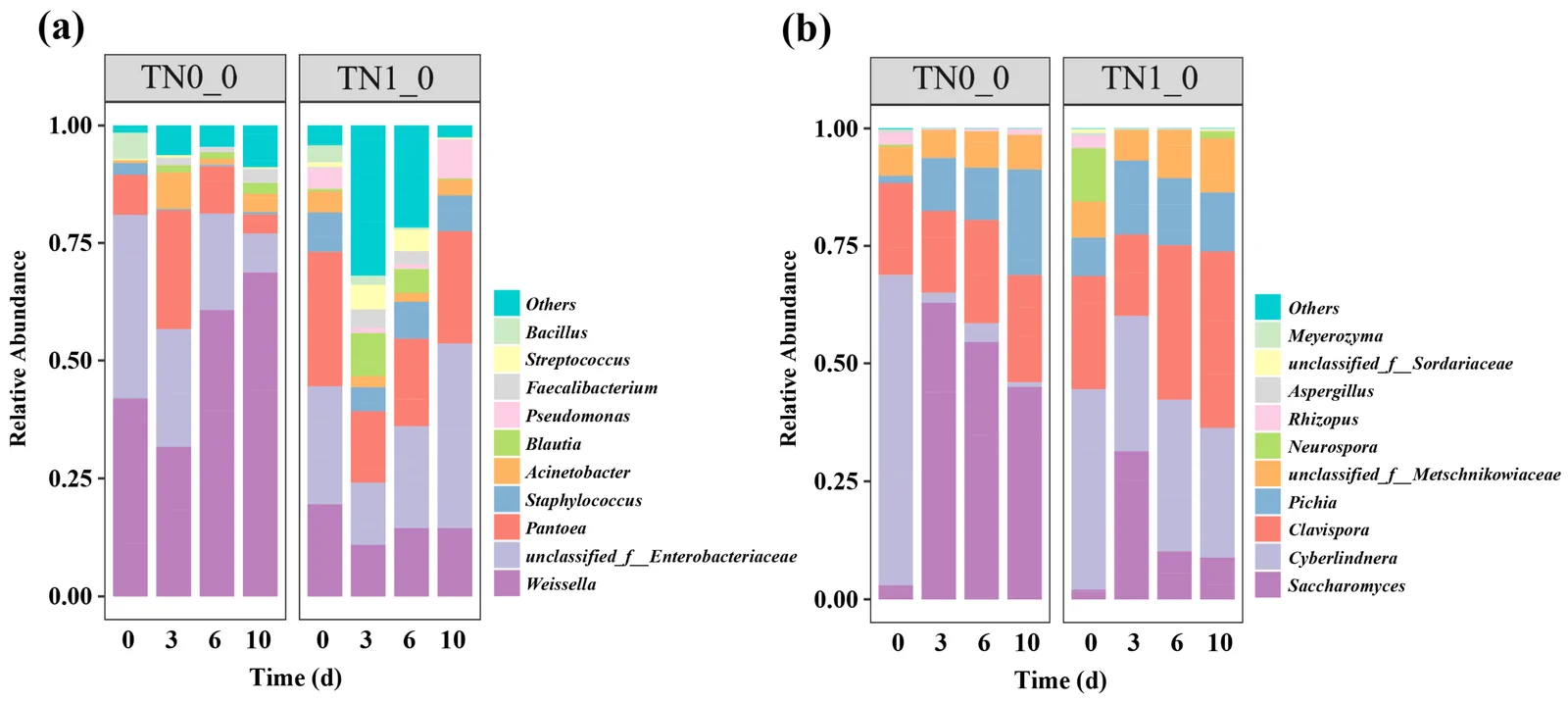
Relative abundance of the top 10 bacterial (**a**) and fungal (**b**) genera in jiupei from TN0_0 and TN1_0 groups at days 0, 3, 6, and 10.

The top 10 bacterial genera with relatively high abundance in the two groups TN0_0 and TN1_0 of jiupei were listed in Figure 2a, including *Weissella*, *unclassified_f__Enterobacteriaceae*, *Pantoea*, *Staphylococcus*, *Acinetobacter*, *Bacillus*, *Pseudomonas*.

Analysis of the TN0_0 group revealed a clear taxonomic succession. While *Weissella* (37.06%) and *unclassified_f__Enterobacteriaceae* (35.35%) showed the highest relative abundances at day 0, the relative abundance of *Weissella* abundance surged to 61.61% by day 10, making it the predominant genus. Conversely, the relative abundance of *unclassified_f__Enterobacteriaceae* declined from 21.89% to 7.81% over the same period. The marked enrichment of *Weissella* in the TN0_0 group is generally consistent with its reported role as an early and mid-fermentation lactic acid bacterium in cereal-based fermentations, where it participates in carbohydrate conversion, acidification, and interspecies interactions [44,45]. A similar pattern was observed for *Pantoea*, whose relative abundance reached 21.89% on day 3 before shrinking, consistent with its recognized role as an early-stage specialist that is gradually replaced as fermentation proceeds [46,47].

In the TN1_0 group, the bacterial community structure differed markedly. *Weissella* showed fluctuating relative abundances and failed to become dominant. *Pantoea* and *unclassified_f__Enterobacteriaceae* exhibited a non-monotonic trend, with their relative abundances decreasing initially before increasing, but both remained below 50% at the end of fermentation. Compared with TN0_0, this weakened dominance of individual bacterial taxa suggests that tannin supplementation imposed a stronger ecological selection pressure and prevented the rapid expansion of a single bacterial genus. Such a pattern is in line with previous observations that phenolic compounds can selectively inhibit sensitive bacteria by interacting with cell envelopes, extracellular enzymes, and nutrient utilization processes, thereby reshaping bacterial succession rather than uniformly suppressing total growth [48]. These findings demonstrate that increased tannin supplementation alters the temporal dynamics of the bacterial community.

Among the fungal genera with relative abundance greater than 1% and ranked in the top 10 (Figure 2b): *Saccharomyces*, *Cyberlindnera*, *Clavispora*, *Pichia*, *unclassified_f__Metschnikowiaceae*, *Neurospora* and *Rhizopus*. Fungal analysis of the jiupei on day 0 identified *Cyberlindnera* as the dominant genus in both TN0_0 and TN1_0, with relative abundances of 65.69% and 42.34%, respectively. A key difference was the significantly higher relative abundance of *Pichia* in TN1_0 (8.18%) compared to TN0_0 (1.58%). This enrichment of *Pichia* at the initial stage may indicate a stronger involvement of non-*Saccharomyces* yeasts in substrate turnover under tannin supplementation, as *Pichia* have frequently been associated with the degradation of complex substrates and the formation of aroma precursors in spontaneous and starter-driven fermentations [49,50]. As fermentation progressed to day 3, the relative abundance of *Saccharomyces* rose significantly to 62.86% and 31.31% in the two jiupei groups. This trend corroborated the study by Ni et al. [51], which documented a community succession from a saccharification-focused phase to one dominated by alcoholic fermentation, heralding the onset of the main fermentative stage. However, the markedly lower abundance of *Saccharomyces* in TN1_0 than in TN0_0 also agrees with the general view that tannins can restrict the competitive expansion of ethanol-dominant yeasts, although the extent of inhibition may vary with fermentation system, tannin level, and microbial background [52].

Throughout fermentation, TN0_0 jiupei was consistently dominated by *Saccharomyces*, while TN1_0 underwent a successional shift toward dominance by non-*Saccharomyces* yeasts (*Clavispora* and *Cyberlindnera*). This suggested that 1.0% tannin may indicate a contribution from a lower relative abundance of *Saccharomyces* but higher relative abundances of alternative yeasts. Consequently, this microbial restructuring may have contributed to the enhanced ester production observed in TN1_0, and, together with the bacterial changes described above, may be associated with a shift in fermentation characteristics toward greater acid and ester accumulation.

#### 3.1.3. Identification of Differentially Abundant Microbes

**Figure 3:**
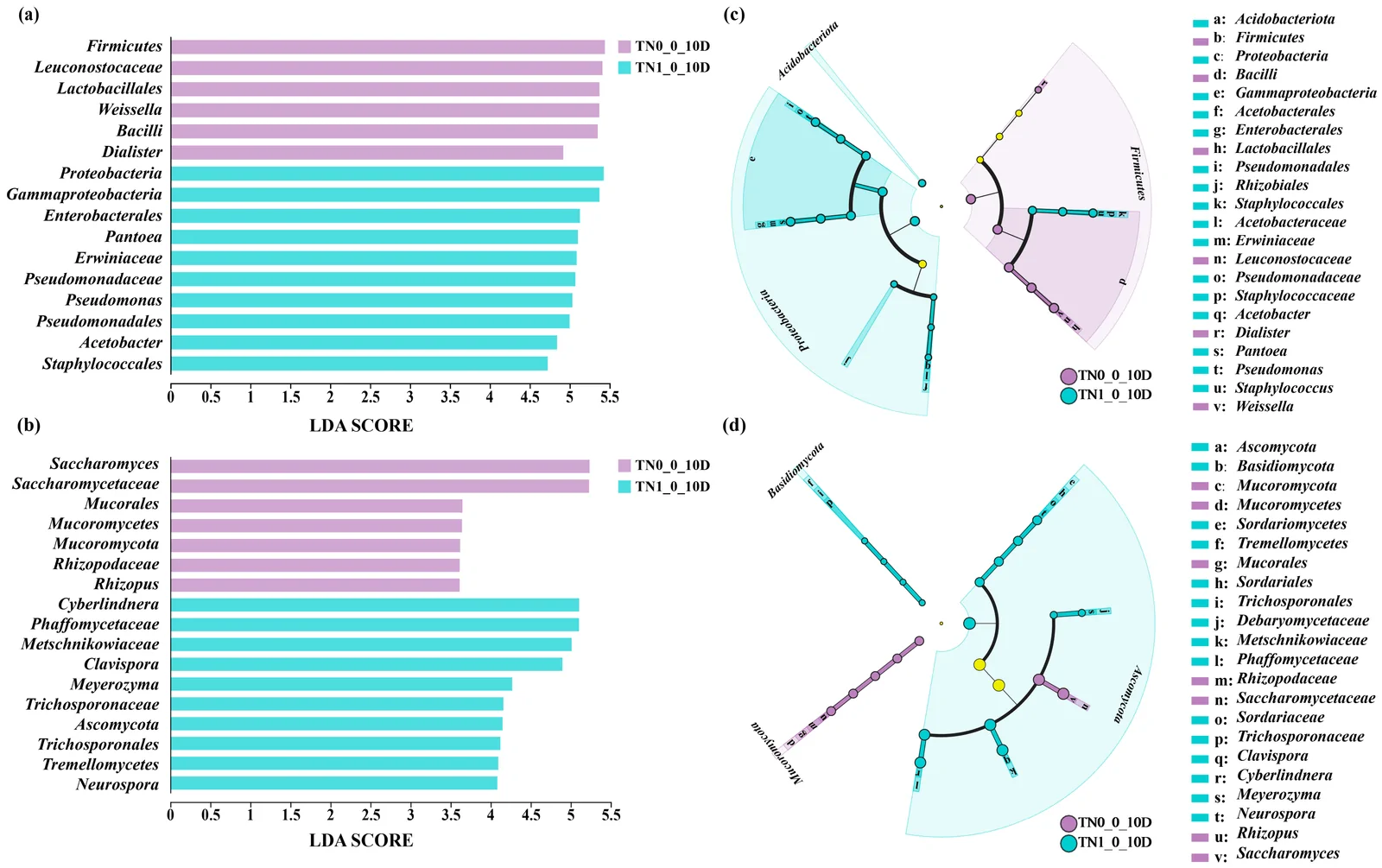
Linear discriminant analysis Effect Size (LEfSe) identifying differentially abundant microbial taxa between TN0_0 and TN1_0 (LDA score threshold of >3.5). Histogram of significantly bacterial taxa (**a**) and fungal taxa (**b**). Cladogram of significantly bacterial taxa (**c**) and fungal taxa (**d**).

The observed successional patterns suggested that tannins exerted a distinct selective pressure, favoring certain non-*Saccharomyces* yeasts and specific bacteria. To identify taxa that statistically discriminated the TN1_0 group from the control, Linear discriminant analysis Effect Size (LEfSe) was employed to screen for candidate microbial biomarkers. LEfSe was employed to identify differentially abundant taxa, using a linear discriminant analysis (LDA) score threshold of >3.5 to highlight taxa with stronger discriminatory effect sizes between groups. The analysis revealed 6 and 10 differentially abundant bacterial taxa in the TN0_0 and TN1_0 groups, respectively (Figure 3a), and 7 and 10 fungal taxa, respectively (Figure 3b). Differentially abundant microbial taxa were identified in both jiupei groups, spanning multiple taxonomic levels from phylum to genus.

The bacterial community structures of TN0_0 and TN1_0 were defined by differentially abundant microbes (Figure 3a,c). TN0_0’s signature taxa, including *Lactobacillales* and *Weissella*, suggested a community oriented towards acidogenesis, with potential implications for substrate acidification and flavor development [45]. Conversely, the TN1_0 consortium was distinguished by the high relative abundance of *Pantoea* (LDA score = 5.10158), establishing it as the most discriminative candidate biomarker. Previous studies have associated *Pantoea* with fermentation-related and flavour-related processes in XLB [53], but in the present study, its enrichment is interpreted primarily as a taxonomic feature distinguishing TN1_0 from TN0_0. Furthermore, other differentially abundant microbes were identified, including *Pseudomonas*, *Acetobacter*, and *Staphylococcus*. Among them, *Acetobacter* has frequently been associated with oxidative metabolism and acid formation in fermentation environments, suggesting that its enrichment may be relevant to the altered metabolic profile of TN1_0 [54]. Overall, these results indicate that tannin supplementation reshaped the bacterial community structure during fermentation and increased the number of taxa contributing to group discrimination.

For the fungal community (Figure 3b,d), the TN0_0 group was predominantly clustered within branches associated with *Saccharomyces* and *Rhizopus*. Conversely, TN1_0 exhibited a distinct fungal signature characterized by a consortium of *Cyberlindnera*, *Clavispora*, *Meyerozyma*, and *Neurospora*. The larger node sizes of these taxa in the cladogram indicate a substantially greater contribution to the observed inter-group dissimilarity. By comparing the two groups of fungal communities, *Saccharomyces* in TN0_0 had a greater impact on the difference effect, which might result in higher alcohol yield in TN0_0 than in TN1_0. Non-*Saccharomyces* yeasts *Cyberlindnera* and *Clavispora* have been reported to possess versatile enzymatic activities and may contribute to early substrate transformation and ester formation, thereby potentially shaping the jiupei flavor profile [55]. These findings suggest that 1.0% tannin supplementation was linked to a lower relative abundance of *Saccharomyces* and a relative enrichment of non-*Saccharomyces* yeasts, particularly *Cyberlindnera* and *Clavispora*. This microbial restructuring may be related to the flavor differences observed between TN1_0 and TN0_0.

### 3.2. The Correlation Among Microorganisms in the Microbial Community

**Figure 4:**
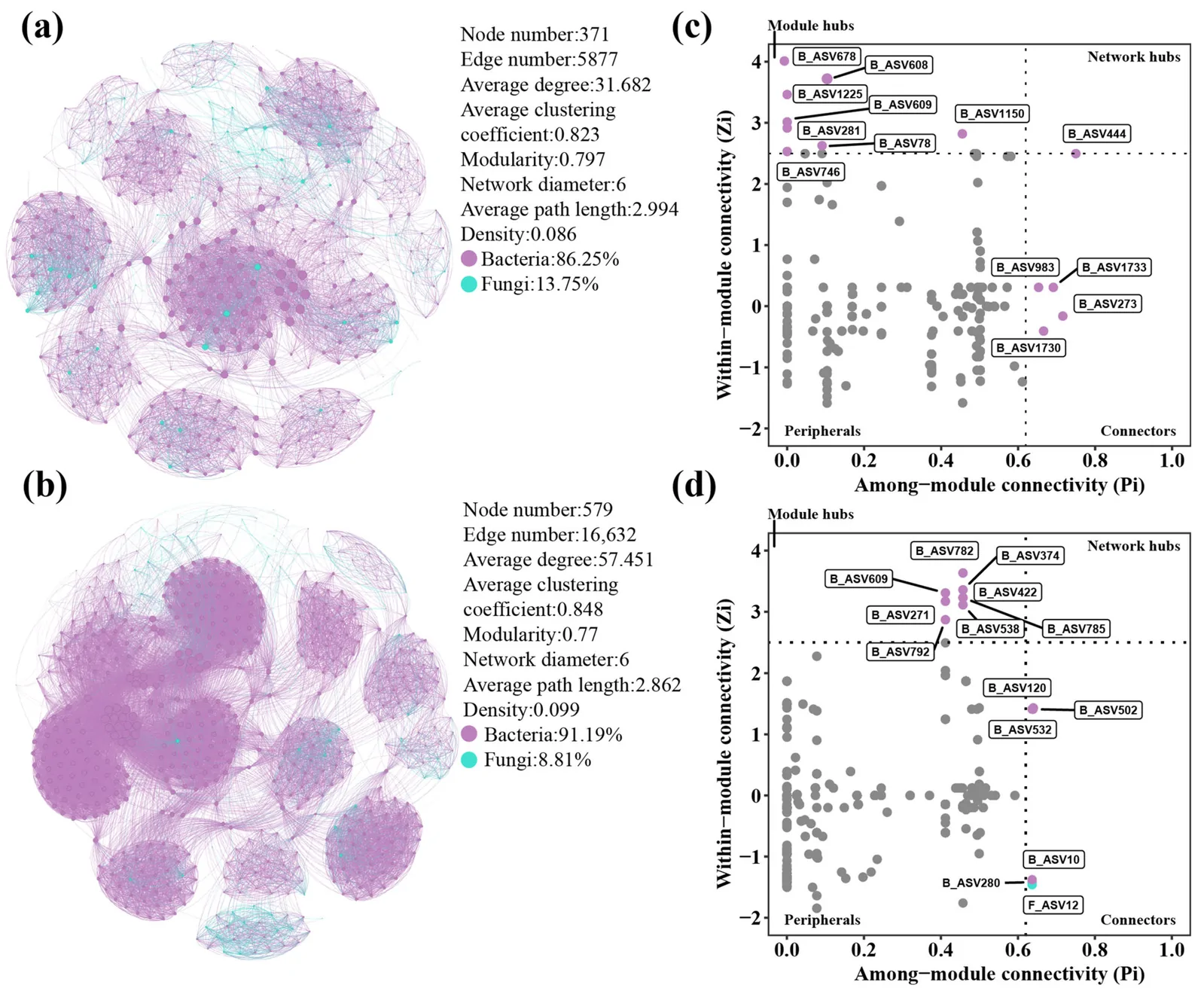
Co-occurrence network analysis of microbial community between TN0_0 (**a**) and TN1_0 (**b**). Topological roles network of microbial community between TN0_0 (**c**) and TN1_0 (**d**). The networks were constructed based on significant microbial correlations, with correlation coefficients screened using |*r*| > 0.6 and *p* < 0.05 as thresholds.

The organization of a fermentation ecosystem may be partially reflected by interspecific association patterns rather than by individual abundances alone, although the resulting networks represent correlation-based relationships rather than direct ecological interactions. In order to assess the effect of tannin supplementation on microbiome associations within the microbial community of jiupei, co-occurrence networks of bacteria and fungi were constructed for TN0_0 and TN1_0, respectively (Figure 4a,b). The networks were constructed based on significant microbial correlations, with correlation coefficients screened using |*r*| > 0.6 and *p* < 0.05 as thresholds. Comparing the co-occurring networks of the two sample groups showed that TN1_0 possessed substantially more network nodes and links than TN0_0. Specifically, the node count in TN1_0 was 1.56 times that of TN0_0, while the link count was 2.83 times greater, suggesting a denser correlation structure among microorganisms in TN1_0 [56]. The average degree and average clustering coefficient of TN0_0 (31.682 and 0.823, respectively) were lower than those of TN1_0 (57.451 and 0.848). Broadly speaking, elevated values of these indices denote intensified node associations and enhanced inter-node connectivity, signifying the network’s overall cohesion and integration [57]. The network was more modular in TN0_0 than in TN1_0, suggesting that the inter-module connections and microbial cross-module associations in TN1_0 increased. With the same maximum transmission distance of the network, the higher the average transmission distance value, the lower the stability of the network [58]. Both network diameters were 6, while the average path length of TN0_0 was higher than that of TN1_0, indicating that the correlation network of TN1_0 may have relatively higher topological stability [59]. Altogether, these results suggest that the TN1_0 network exhibited greater complexity and a more tightly connected topological structure than TN0_0. In this context, tannin supplementation was associated with a shift in microbial correlation patterns within jiupei.

Divergence in the topological roles of individual member species within TN0_0 and TN1_0 networks was determined using within-module (Zi) and among-module (Pi) connectivity metrics, elucidating topological disparities at the species level (Figure 4c,d). Calculating the Zi and Pi of nodes, nodes were distributed across four distinct sections of the topological roles network: Module hubs, Network hubs, Connectors, and Peripherals [60]. The nodes named keystone nodes were divided into Module hubs, Network hubs, and Connectors. The TN0_0 network contained 13 keystone nodes, all of which were bacterial nodes [61]. A total of 14 nodes, including 1 fungi node, could be considered as keystone nodes in the network of TN1_0. All these keystone nodes representing microorganisms didn’t overlap. Therefore, the TN0_0 and TN1_0 networks appeared to differ in their topological organization at the keystone-node level, a pattern that may be related to tannin supplementation.

### 3.3. Volatile Flavor Composition of Jiupei and Its Association with Microbial Communities

The distinct microbial community structures observed between TN0_0 and TN1_0 were accompanied by differences in volatile composition, suggesting that tannin supplementation may be associated with altered metabolic output in jiupei. Rather than evaluating direct sensory consequences, this section focuses on the compositional differences in volatile flavor compounds present in jiupei after fermentation, which may also provide an indication of compounds with potential relevance for subsequent liquor aroma. Therefore, volatile flavor compounds in jiupei after 10 days of fermentation were profiled by HP-SPME-GC-MS, and their associations with differentially abundant microbial taxa were further examined.

#### 3.3.1. Comparison of Volatile Flavor Compounds in Jiupei

**Figure 5:**
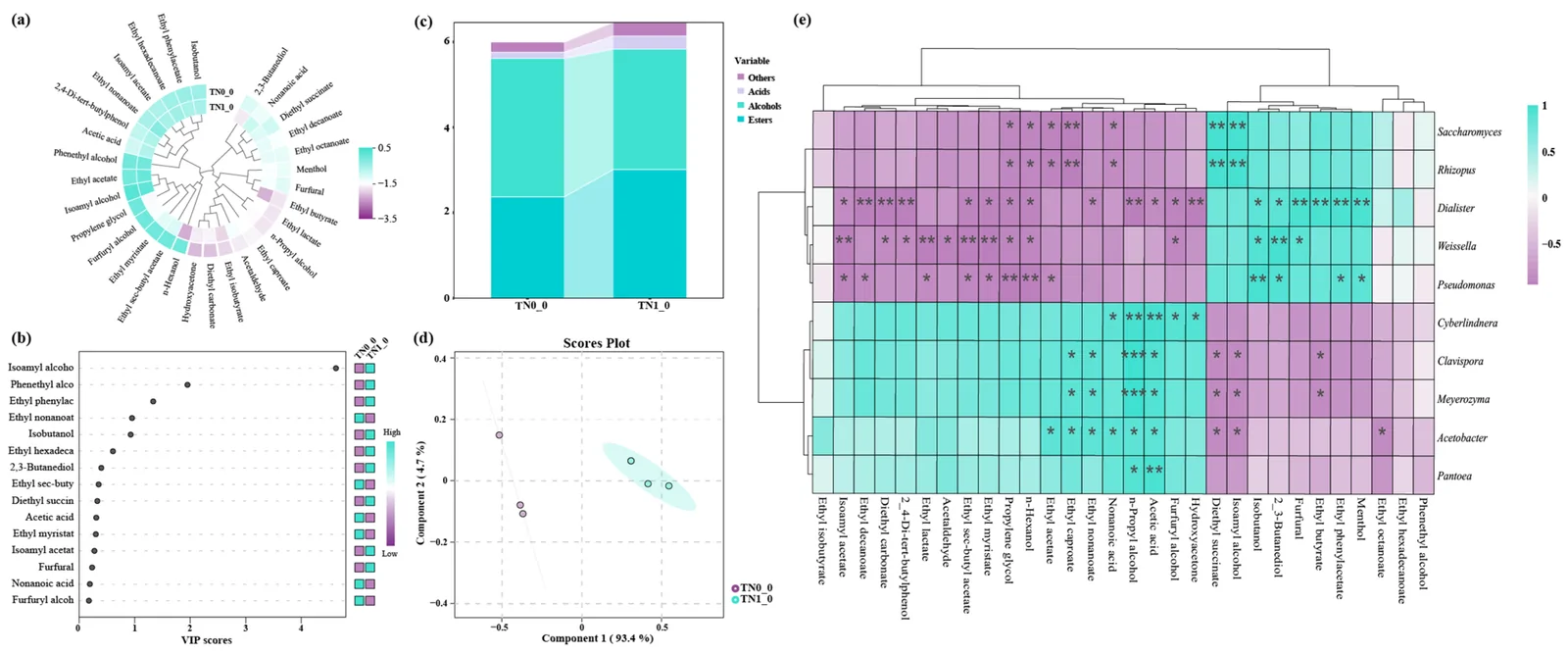
Volatile flavor compounds of jiupei. (**a**) Heat map of volatile matter content of two groups of jiupei. (**b**) VIP values of key aroma components. (**c**) Stacked plot of volatile matter categories of jiupei. (**d**) Score of OPLS-DA samples for jiupei aroma. (**e**) Heat map of the correlation between main flavor substances and different microorganisms. Asterisks (*, **, ***) denote the significance levels after false discovery rate (FDR) correction (*p* < 0.05, *p* < 0.01, *p* < 0.001).

A total of 30 volatile flavor compounds were detected across TN0_0 and TN1_0 on the 10th fermentation day (Figure 5a–d), including 25 compounds in TN0_0 (total content: 4.208 μg/g) and 30 compounds in TN1_0 (total content: 4.983 μg/g). TN0_0 jiupei contained 13 esters, 6 alcohols, 2 acids, along with 4 supplementary volatile flavor compounds. TN1_0 jiupei contained 15 esters, 9 alcohols, 2 acids, and 4 other volatile flavor compounds. The ester content of TN1_0 (3.01 μg/g) significantly surpassed that of TN0_0 (2.37 μg/g), with special esters like Ethyl acetate and Ethyl nonanoate standing out. Among the esters showing notable differences, ethyl acetate and ethyl caproate are widely recognized as the major volatile compounds in XLB [62]. Therefore, changes in these compounds in jiupei may be relevant to the aroma potential of the final Baijiu if they are retained or transferred during subsequent processing. This difference may be associated with the altered microbial structure of TN1_0, particularly the relative enrichment of non-*Saccharomyces* yeasts, which could influence precursor availability and ester-related metabolism. In particular, the lower relative abundance of *Saccharomyces* together with the enrichment of genera such as *Cyberlindnera* and *Clavispora* is consistent with a fermentation system in which ester formation may depend more strongly on non-*Saccharomyces* substrate transformation and on the availability of organic acid precursors, rather than on *Saccharomyces* alcohol production alone [63]. The alcohol content of TN1_0 (2.81 μg/g) was decreased by 13.3% compared to TN0_0 (3.24 μg/g). This pattern may be related to differences in fungal community composition between TN0_0 and TN1_0, since TN0_0 was characterized by a stronger contribution of *Saccharomyces*. The acid content of TN1_0 was nearly twice as much as that of TN0_0. This observation was consistent with the higher relative abundance of *Staphylococcus* in TN1_0, as *Staphylococcus* has been reported to be related to the synthesis of substances such as acetic acid, butyric acid, and heptanoic acid [64]. The same applies to supplementary volatile flavor compounds, which included acetaldehyde, furfural, hydroxyacetone, and 2,4-di-tert-butylphenol, and were detected in both TN0_0 and TN1_0, while their contents were different.

To further evaluate the potential odor relevance of these compositional differences, odor activity values (OAVs) were calculated using odor thresholds from the literature (Table S1). OAV analysis revealed that nonanoic acid, ethyl caproate, and ethyl phenylacetate exhibited OAV > 1 in both groups, with nonanoic acid increasing markedly in TN1_0, whereas none of the detected alcohols reached an OAV ≥ 1, consistent with the higher acid and ester accumulation and lower alcohol abundance in the TN1_0 group.

Although some of the major esters showing differences between groups are recognized as important flavor compounds in XLB, the present data were obtained from jiupei rather than the final Baijiu. Therefore, these compositional differences are more appropriately interpreted as indications of altered fermentation chemistry and possible aroma transfer potential, rather than as direct evidence of sensory impact. In addition, because the HP-SPME-GC-MS results were semi-quantitative, relatively small differences in compound abundance should be interpreted with caution.

In summary, tannin supplementation was associated with differences in the composition and relative abundance of volatile flavor compounds in jiupei after 10 days of fermentation, including higher ester richness, lower alcohol abundance, and greater acid accumulation in TN1_0. These trends may be related to the observed shift from a *Saccharomyces*-dominated fungal pattern toward a community with greater representation of non-*Saccharomyces* yeasts and acid-associated bacteria, although the underlying metabolic contributions require further verification.

#### 3.3.2. Associations Between Differentially Abundant Microbes and Flavor Compounds

Differences in ester and acid abundance between the tannin-supplemented and control groups suggested that the microbial shifts observed in jiupei might be accompanied by changes in volatile composition. To elucidate the potential relationships between the microbial community and flavor compounds, a Spearman correlation analysis was conducted between the differentially abundant microbial genera and the volatile flavor compounds identified in the jiupei. The resultant heatmap (Figure 5e) revealed a complex and significant correlation network. The color spans from deep purple (negatively correlated) to light blue (positively correlated). The numerical values range from −1 to 1. Asterisks (*, **, ***) denote the significance levels after false discovery rate (FDR) correction (*p* < 0.05, *p* < 0.01, *p* < 0.001). Spearman correlation analysis reflects statistical associations rather than causative relationships. As noted above, Spearman correlation reflects statistical association rather than direct causation; therefore, the results should be interpreted as potential links between microbial abundance and volatile compounds rather than direct metabolic relationships.

The differentially abundant microbes of TN0_0, namely *Saccharomyces*, *Rhizopus*, *Dialister*, and *Weissella*, were predominantly linked to an alcohol-oriented correlation pattern. Notably, both *Saccharomyces* and *Rhizopus* showed significant positive correlations with diethyl succinate and isoamyl alcohol, yet surprising negative correlations with key esters like ethyl acetate. This pattern may be consistent with the comparatively alcohol-associated volatile profile of TN0_0 and with the stronger relative contribution of *Saccharomyces* in this group. Furthermore, the extensive negative correlations exhibited by *Dialister* and *Weissella* with a broad spectrum of esters (e.g., ethyl lactate, ethyl decanoate, ethyl caproate) suggest that these genera were associated with a less ester-enriched volatile pattern in TN0_0.

In stark contrast, the microbial consortium characteristic of TN1_0, including *Cyberlindnera*, *Clavispora*, *Meyerozyma*, *Acetobacter*, and *Pseudomonas*, showed an association pattern broadly consistent with the enhanced ester and acid content observed in this group. *Clavispora* and *Pseudomonas* consistently showed significant positive correlations with esters such as ethyl nonanoate, ethyl caproate, and ethyl phenylacetate. Given that non-*Saccharomyces* yeasts are often associated with flavor diversification and precursor transformation during fermentation, the positive correlations of *Clavispora* and *Cyberlindnera* with multiple esters may indicate their potential relevance to ester-associated processes in TN1_0. More importantly, *Acetobacter* and *Pantoea* were strongly positively correlated with acetic acid and n-propanol. Because acetic acid can serve as a substrate for esterification, the enrichment of *Acetobacter* and its positive association with both acetic acid and ethyl acetate is consistent with a fermentation environment that may favor acetate ester formation. The significant positive correlation of *Cyberlindnera* and *Meyerozyma* with acetic acid and nonanoic acids also corresponded to the higher acid content in TN1_0.

In conclusion, the correlation analysis provided association-based evidence for understanding the distinct volatile profiles. The TN0_0 was characterized by a microbiota (*Saccharomyces*, *Rhizopus*, *Dialister*, *Weissella*) whose correlative pattern was more closely associated with certain alcohols and with comparatively lower ester abundance. Conversely, the TN1_0 showed a microbial association pattern involving non-Saccharomyces yeasts and acid-associated bacteria, including *Cyberlindnera*, *Clavispora*, *Meyerozyma*, and *Acetobacter*, that was consistent with higher ester and acid abundance. This consortium showed positive correlations with acid precursors, which may help elucidate the greater diversity and abundance of esters observed under the 1.0% tannin supplementation.

### 3.4. Metabolic Functional Potential of Microbial Communities

The observed correlations between specific bacteria and esters suggest potential associations between microbial succession and flavor-related metabolite changes; however, the underlying biological mechanisms remain shrouded. To further explore the potential functional basis of these patterns, we inferred bacterial metabolic functional potential from 16S rRNA gene ASV profiles using PICRUSt2, with functional annotations summarized according to the Clusters of Orthologous Groups database, whereas fungal ecological trophic modes were assigned from ITS ASV profiles using FUNGuild [65]. These analyses represent taxonomically inferred functional or ecological potential rather than directly measured metabolic activity.

**Figure 6:**
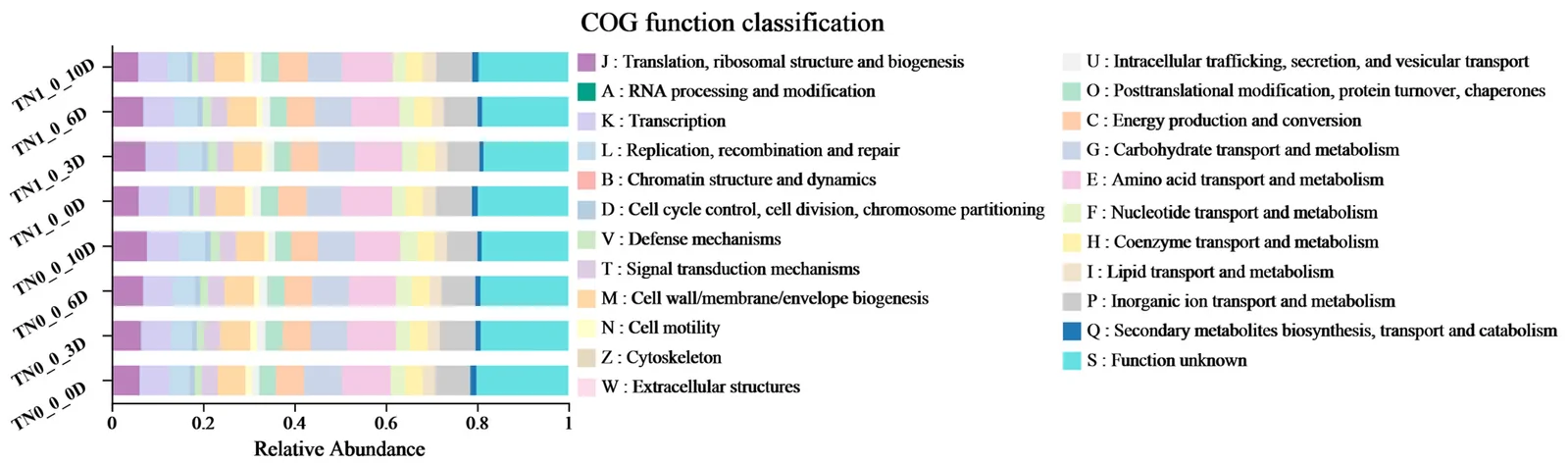
Predicted metabolic functional potential of microbial communities in XLB jiupei from TN0_0 and TN1_0 groups at days 0, 3, 6, and 10, based on COG annotation.

As shown in Figure 6, the PICRUSt2 functional profiles revealed a consistent enhancement in predicted metabolic potential in TN1_0 compared to TN0_0 across the fermentation timeline. This prediction was consistent with the previously observed microbial structural shifts, particularly the enrichment of taxa related to carbon utilization and flavor precursor formation in TN1_0. This trend is broadly consistent with the compositional shifts observed in TN1_0, although functional inference from taxonomic profiles should be interpreted with caution.

A relatively higher predicted capacity was inferred for TN1_0 in Category G, carbohydrate transport and metabolism, indicating enhanced potential for substrate utilization. This functional predicted enrichment may be related to the enrichment of genera such as *Pantoea*, which have been reported to possess saccharolytic capabilities [66]. Concurrently, substantially elevated predicted functional representation was observed in Category E, amino acid transport and metabolism, which may increase the availability of amino acid-derived precursors relevant to flavor formation. Significantly increased predicted functions in Category E (amino acid transport and metabolism) may enhance the availability of amino acid precursors for flavor formation [8]. Sustained enhancement was shown by Category C, energy production and conversion pathways, throughout fermentation, suggesting a greater predicted capacity for energy metabolism in TN1_0. Furthermore, a significant increase in representation was noted in Category H, coenzyme transport and metabolism, which may support cofactor-dependent biochemical transformations during fermentation. Category I (lipid transport and metabolism) and Category P (inorganic ion transport and metabolism) were also relatively enriched in TN1_0. Since lipid metabolism is closely related to fatty acid turnover and membrane-associated metabolic processes, these predicted functional differences may be relevant to the formation of acid and ester precursors. In particular, lipid-related metabolism may influence the supply and transformation of fatty acid precursors that are important for ester biosynthesis during Baijiu fermentation.

Taken together, the enrichment of Categories G, E, C, H, and I suggests that TN1_0 possessed a broader predicted functional capacity for carbohydrate conversion, amino acid turnover, energy metabolism, cofactor utilization, and lipid-related metabolism. These functions are all potentially relevant to the generation of alcohols, acids, and ester precursors during fermentation. Notably, several ester compounds that changed significantly in this study, such as ethyl acetate and ethyl phenylacetate, are widely recognized as important aroma contributors in XLB [3]. Ethyl acetate is generally associated with fruity and clean aroma notes in light-flavor Baijiu, whereas ethyl phenylacetate is often linked to floral and honey-like sensory nuances [62]. It should be noted that PICRUSt2-based inference reflects only the predicted metabolic potential of bacterial communities and does not directly capture gene expression, enzyme activity, or actual metabolic flux. Therefore, the functional interpretations proposed here should be considered preliminary and require further experimental validation.

Regarding the fungal communities, FUNGuild analysis revealed differences in the predicted trophic-mode composition between TN1_0 and TN0_0 throughout fermentation (Figure S1). Saprotrophic and saprotroph-containing mixed trophic modes represented important components of the fungal functional profiles, suggesting that the fungal communities possessed potential ecological capacities related to the utilization and transformation of grain-derived organic substrates [67,68]. These predicted functions may facilitate the degradation of complex substrates and the release of fermentable sugars, amino acids, fatty acids, and other flavor precursors during solid-state fermentation [69].

The differences in fungal trophic-mode composition were broadly consistent with the succession of non-*Saccharomyces* fungi and the observed shift in volatile flavor profiles. In TN1_0, the enrichment of non-*Saccharomyces* fungi associated with acids and esters coincided with changes in saprotrophic and saprotroph-containing functional groups, suggesting a potentially greater fungal contribution to substrate conversion and precursor availability. Such changes may favor the formation of organic acids and their subsequent esterification, whereas the fungal community in TN0_0 was more closely associated with an alcohol-oriented flavor profile. Taken together, the FUNGuild results suggest that tannin supplementation was associated with changes in the predicted ecological functions of the fungal community, particularly those related to saprotrophic substrate utilization.

The coordinated enhancement of these core metabolic functions, combined with previously documented microbial succession toward a community enriched with functional specialists, suggests that tannin supplementation may have contributed to coordinated shifts in microbial structure and predicted metabolic capacity.

## 4. Conclusions

This study showed that 1.0% tannin supplementation was associated with changes in microbial community structure and flavor profile during XLB fermentation. Tannin supplementation was generally associated with increased bacterial richness and diversity, altered bacterial and fungal communities, reduced the relative dominance of *Saccharomyces* and *Weissella*, and enriched several non-*Saccharomyces* yeasts and bacterial genera, including *Cyberlindnera*, *Clavispora*, *Pantoea*, and *Pseudomonas*. The tannin-supplemented group also exhibited a more complex microbial co-occurrence network. Meanwhile, the volatile profile was characterized by the fact that total esters and acids increased by 27.0% and 112.3%, respectively, whereas total alcohols decreased by 13.3%. Correlation analysis further identified potential associations relevant to flavor modulation, indicating that tannin supplementation was related to the microbial community towards a consortium of non-*Saccharomyces* fungi and acid-producing bacteria. These microorganisms were strongly associated with acids and esters, suggesting that the altered microbial community and precursor availability may have contributed to the greater diversity and abundance of esters. PICRUSt2-based prediction suggested relatively enhanced functional potential related mainly to carbohydrate, amino acid, and energy metabolism. FUNGuild analysis suggested altered fungal saprotrophic functions under tannin supplementation. However, these findings are based on predicted functions and correlation-based analyses; therefore, causal mechanisms require further validation. In addition, because volatile compounds were measured in jiupei rather than in the final distilled Baijiu, their sensory relevance remains to be confirmed. Overall, 1.0% tannin supplementation may help improve XLB fermentation quality and provide practical insights into the rational utilization of tannin-containing sorghum for producing XLB.

## Data Availability

The original contributions presented in this study are included in the article/Supplementary Materials. Further inquiries can be directed to the corresponding authors.

## Supplementary Materials

The following supporting information can be downloaded at: https://www.mdpi.com/article/10.3390/foods15162833/s1, Table S1. Odor activity values (OAVs) of volatile compounds identified in jiupei (TN0_0 and TN1_0). Figure S1. FUNGuild-based functional prediction of fungal communities.

## Abbreviations

The following abbreviations are used in this manuscript:

XLB	Xiaoqu light-flavor Baijiu
ASVs	Amplicon sequence variants
HS-SPME-GC–MS	Headspace solid-phase microextraction gas chromatography–mass spectrometry
OAVs	Analyzing odor activity values
EI	Electron ionization
RIs	Retention indices
PCoA	Principal coordinate analysis
LEfSe	Linear discriminant analysis effect size
LDA	Linear discriminant analysis
