# Supplementary material for: Regulatory Effects of Tannin Supplementation on Microbial Succession and Flavor Formation During Xiaoqu Light-Flavor Baijiu Fermentation
Source: Foods. 2026 Aug 14;15(16):2833. doi: 10.3390/foods15162833 (PMC13512506; doi:10.3390/foods15162833)
Supplement: Supplementary file 1 [file foods-15-02833-s001.zip › foods-4458864-supplementary.pdf]

Supporting information

Table S1. Odor activity values (OAVs) of volatile compounds identified in jiupei (TN0_0 and TN1_0).

No.	Compound	Odor Threshold (μg/g)	Conc. in TN0_0 (μg/g)	OAV-TN0_0	Conc. in TN1_0 (μg/g)	OAV-TN1_0
1	Isoamyl alcohol	7.000	1.787	0.255	1.472	1
2	Phenethyl alcohol	14.000	0.975	0.070	0.931	0.066
3	Ethyl acetate	7.500	0.877	0.117	1.178	0.157
4	Ethyl phenylacetate	0.073	0.385	5.271	0.245	3.354
5	Isobutanol	75.000	0.333	0.004	0.261	0.003
6	Ethyl hexadecanoate	0.007	0.326	46.508	0.318	45.494
7	Ethyl nonanoate	12.000	0.267	0.022	0.573	0.048
8	Isoamyl acetate	1.600	0.236	0.148	0.266	0.167
9	2,4-Di-tert-butylphenol	330.000	0.143	0.000	0.216	0.001
10	Diethyl succinate	200.000	0.101	0.001	0.068	0.000
11	Acetic acid	5.600	0.099	0.018	0.203	0.036
12	2,3-Butanediol	150.000	0.077	0.001	0.020	0.000
13	Furfural	0.068	0.065	0.965	0.039	0.585
14	Ethyl octanoate	0.900	0.058	0.064	0.056	0.063
15	Menthol	5.000	0.052	0.010	0.036	0.007
16	Nonanoic acid	0.00071	0.048	67.021	0.107	150.862
17	Ethyl decanoate	0.200	0.037	0.183	0.058	0.288
18	Ethyl caproate	0.005	0.026	5.119	0.031	6.240
19	Acetaldehyde	0.020 [2]	0.021	1.043	0.041	2.053
20	Ethyl butyrate	0.020	0.018	0.917	0.006	0.312
21	Ethyl lactate	14.000	0.018	0.001	0.031	0.002

22	n-Propyl alcohol	0.094	0.018	0.192	0.028	0.296
23	Ethyl isobutyrate	0.015	0.013	0.886	0.014	0.905
24	Diethyl carbonate	n.d.	0.009	n.d.	0.024	n.d.
25	Hydroxyacetone	10.000	0.007	0.001	0.021	0.002
26	Ethyl sec-butyl acetate	0.31 ppm	0.000	n.c.	0.075	n.c.
27	Ethyl myristate	4.000	0.000	0.000	0.064	0.016
28	Furfuryl alcohol	8 ppm	0.000	n.c.	0.038	n.c.
29	Propylene glycol	n.d.	0.000	n.d.	0.027	n.d.
30	n-Hexanol	0.006	0.000	0.000	0.006	0.94

Abbreviations: n.d., not determined due to unavailable odor threshold data; n.c., not calculable because the reported threshold is in gaseous phase (ppm, v/v), which cannot be directly converted to liquid/solid matrix concentration (μg/g).

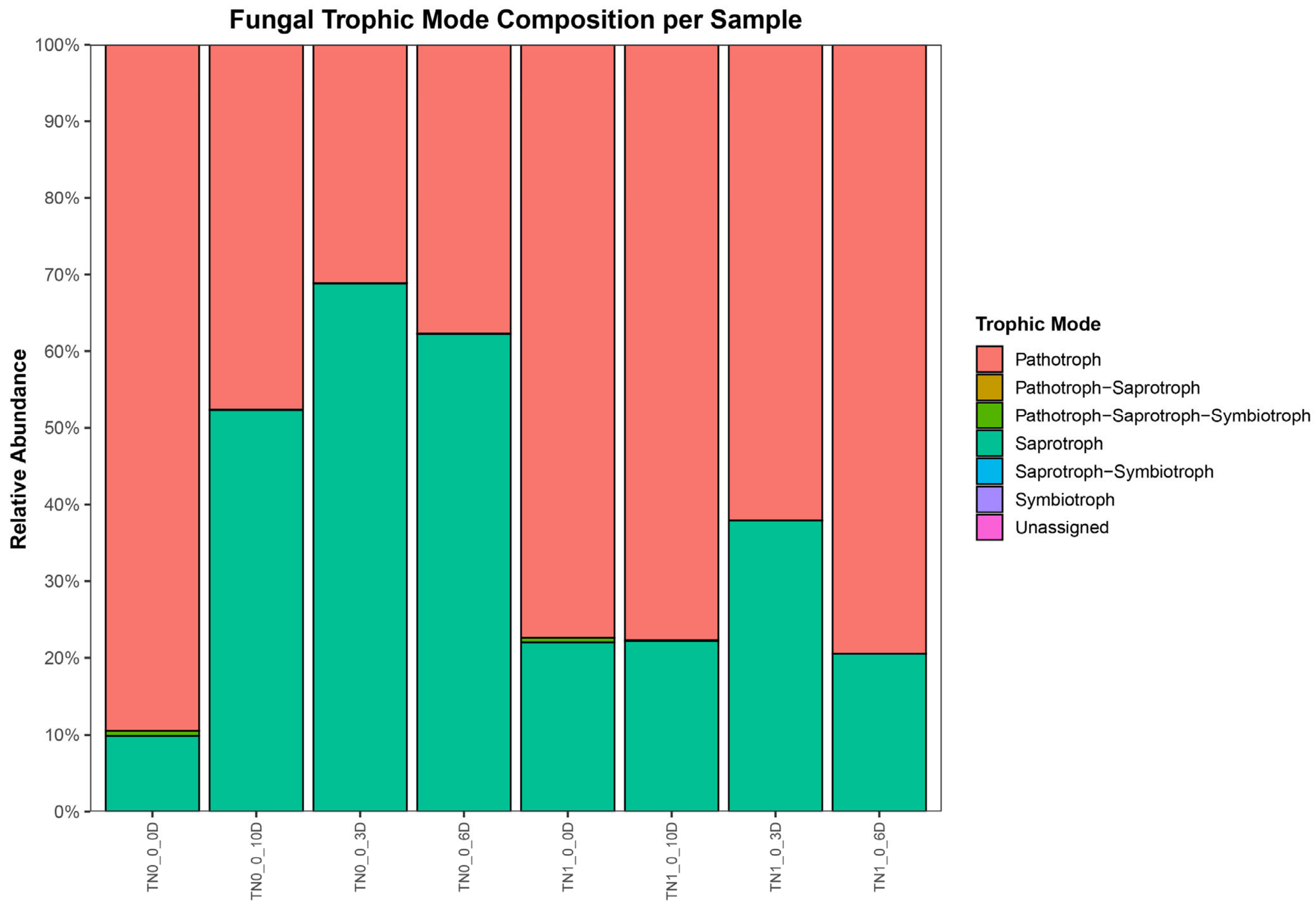

Figure S1. FUNGuild-based functional prediction of fungal communities.
